# Summary and Extracts from a Report of the Lecture Delivered Introductory to the Course in the University of Buffalo

**Published:** 1862-12

**Authors:** James P. White

**Affiliations:** Professor of Obstetrics and Diseases of Women and Children


ART. II.—*Summary and Extracts from a Report of the Lecture delivered introductory to the Course in the University of Buffalo, by* JAMES P. WHITE, M. D., *Professor of Obstetrics and Diseases of Women and Children.*

"GENTLEMEN:

In advanced periods of the cultivation of some of the more important arts of life, the principle of division of labor, has been recognized as useful, and *necessary* towards their *further* improvement and perfection. In the pursuit of medical science the same principle has been acknowledged and made available to the best purposes of medical education. Without neglecting any branch of study, essential to the acquisition of competent medical knowledge, in all its departments; it seems proper that individual members of the profession should undertake to teach particular portions of the general science. On this principle are founded in most of the universities and other great schools, both of Europe and America, certain established arrangements, for assigning to different professors or lecturers, different allotments of duty, corresponding with the various branches of science or literature to be taught. In the greater number of such schools and universities, one of the branches regarded as essential to all aspiring to the honors of the profession is that of midwifery, or obstetric medicine, in its most comprehensive sense.

Thus understood, obstetric medicine may be said to be identified with all the scientific principles and rules of art that can be made applicable, not only to the practice merely of midwifery, as synonymous with the professional services usually rendered the female during parturition, but also to the medical superintendence and treatment of their peculiar functions and diseases. Accordingly, in all the best appointed schools of medicine in modern times, it is made the duty of the professors of midwifery to deliver lectures on the peculiar organs, functions, and diseases of the human female.

Influenced by the considerations just alluded to, the Council of this University, in the organization of the medical department, have created a separate chair, and assigned to me the duty and honor of its occupation. Of the co-ordinate departments in this University and the able manner in which the duties devolving upon my colleagues will be discharged, I can speak without hesitation. Yes, gentlemen, I am happy in being able to assure you that should you not become fully acquainted with the subjects taught by these able men, the responsibility of the failure will rest with you. And on the part of this Institution he assured that it has ever been the deliberate determination of the Faculty of this University to maintain a position second to none in the country. In many things essential to an elevated standard of medical education the founders of this College claim to have *led* the way in reform and improvement. It has ever been also the desire of those to whom the interest of this Institutiou have been confided to send forth such alumni only as would do honor to that profession to which its credentials might form the passport. We prefer to be judged by the *quality* rather than the amount of fruit annually furnished, relying upon the ultimate and permanent attainment of success in this way, rather than temporarily enjoy the ephemeral reputation of cheap lectures, and indiscriminate graduations, and can even now, after a brief existence of fifteen years, with pride point to a long list of honorable names as the representatives of these principles, and with the Roman matron exclaim, "these are our jewels," convinced that by increasing the facilities for a more thorough and efficient course of instruction, as well as by making a high standard of attainment requisite to the reception of its honors, the true interests of the student and school are alike consulted, our course now as heretofore, shall be onward and upward. Satisfied that our success should be measured by our merits, we court impartial comparison with our much respected sister institutions. With unfeigned pleasure do we point to the fact that, of the many applicants for admission to the several departments of the service, not a single rejection of a recipient of the honors of this Institution has come to the knowledge of its Faculty.

But I am digressing, and must return from these more general questions to consideration of the importance of one of the departments—Obstetrics, and the means for its successful prosecution. In the outset then, gentlemen, permit me to say that in the humble effort to lead your minds in the pursuit of obstetric science, and faithfully to conduct you into the rudimental principles of this important province, I would bespeak your generous sympathies, your careful attention, and your untiring industry.

The first is solicited for myself, conscious that with my best efforts much that is imperfect can be detected. Relying rather upon your cordial co-operation than upon any surpassing ability on the part of your teacher; promising to hold myself at all times in readiness, diligently to impart instruction to all who may have the patience or desire to listen, relying mainly upon *your* assiduity, I can have no hesitation in predicting that success must crown our labors. Nor can I entertain any fears that you, gentlemen, can be so blind to your interests, your comfort, or your honors, as not to bestow your careful attention, your utmost diligence in the acquisition of a competent knowledge of a subject which, to you, is of vital importance.

In view of the importance of the subjects which it will become my duty to bring under your consideration, I have thought it might be useful for us to bestow a short time to the preliminary question as to the *manner* in which it could be most profitably pursued. Not that I suppose the manner of acquiring a knowledge of midwifery differs essentially from the best method of making yourselves general practitioners of medicine. Indeed most of the suggestions will be equally applicable in advancing you in any of the departments of medical *science*. All the studies which you are about to commence are attractive in character, affording the amplest range for the exercise of the highest intellectual powers, and are also calculated to develope the best feelings of our nature. Their object contemplates the well-being of our fellow creatures, the lessening of human suffering, and the extension of human existence.

To the full accomplishment of this end, it becomes necessary not only that the student familiarize himself with medicine in its more restricted sense; but the intelligent physician should be acquainted with the various relations of man, with nature, with society, and all external agencies, and with himself; should ascertain their respective influences, trace their actions and define their laws. With so extensive a field for intellectual exertion, can it be a matter of surprise that medicine, in its more liberal acceptation, should be accounted one of the most difficult of studies, so much so indeed that long life will hardly suffice for its complete mastery? Under these circumstances it certainly behooves us to call to our aid all the means and appliances which can further our progress, and sustain us during the labors incident thereto.

In the first place allow me then to say, that in my candid opinion, every student will consult his best interests by pursuing such a preliminary course of studies as will give aptness to his mental faculties, render his perceptions vivid, cultivate his memory, mature his judgment, and discipline his mind to the acquisition of knowledge, and enable him to digest and arrange what he learns. And here permit me to cite in proof of this position, the opinion of the assembled wisdom of the National Medical Association, as repeatedly pronounced, and recommended to your careful consideration the suggestions of that learned body upon this subject. I do not design to be understood as saying that no man *can* become both useful and eminent without this preliminary course of mental training. Experience abundantly proves that industry has often triumphed over all these obstacles with many others superadded. Nor should these studies ever be pursued as ends, but as means, as lights which can illumine the paths on which you are about to enter. When thus disciplined, the mind is better prepared for *intelligent observation.*

And here I would call your attention more particularly to this most difficult of processes, *correct observation.* The facts submitted to the *medical* observer are peculiarly complex, and often difficult satisfactorily to analyze. Besides, what an infinite number of sources of deception. A theory previously formed, with a foregone, but unconscious determination to support it; long established habits, sustained perhaps by the so-called *experience* of our seniors, and ourselves; fondness for, or a determination to resist, the introduction of novelties; these, and many other sources of error may be mentioned, as likely to lead the judgment of the honest observer from the truth. It is clear that there is a right and a wrong method of observing or studying disease. Physicians may be said always to have observed, yet how seldom have they discovered. A question of primary importance to the practitioner of medicine consists then in ascertaining the *true* method of inquiry. It is obvious that simple experience—merely witnessing many cases of the same disease—is by no means certain to conduct us to correct results. One practitioner will treat hundreds of consecutive cases of puerperal fever by copious venesection; whilst his neighbor, seeing an equal number of cases, and being equally conscientious, will as invariably give stimulants or opiates, in the firm conviction that in so doing alone can he save his patients. Our purpose or object in observing should be to make use of facts; and we can be said to possess the power of *using* a *fact*, *only* when we ascertain its *cause*, and *what*, in *its* turn, it will produce. If the fact observed be recognized by the mind as forming a link of a consecutive series, we are said to be in possession of *science*, or *scientific knowledge*. We can there trace its emanation, and its product; in short, we can avail ourselves of it; we can use it.

Again, we must arrange and digest what we observe. It would be easy to show from the consideration of facts, taken in any of the other departments of human knowledge that science, or scientific acquirement can exist *only* where we observe a series of consecutive and connected facts, so that, by having one of them we are enabled to trace its connection, bearing, and effection upon the whole chain.

Our first object and purpose then being the collection of consecutive and connected facts, the best method of its accomplishment will be secured by following the directions of the immortal Bacon. "Man," says this original thinker, "the servant and interpreter of nature, understands and can effect just so much as he has actually experienced—more he can neither know nor achieve." In a word, then, the medical practitioner must *interpret*—not theorize or conjecture. And it is essential here, in limine, in taking the incipient steps in your profession, as well as in your after practice, that you eschew theories, else you will find a constant tendency in the mind to accommodate the *facts* observed to preconceived opinions, instead of forming the judgment, or deducing conclusions from a fair and legitimate analysis, of impartial observation. Can we not in this way account for the difference between the practice of English and continental obstetricians, in the employment of the forceps and the crotchet? Indeed, will not this resolute determination to establish a darling fancy, account for *all* the exclusive theories which have pervaded medicine, and doubtless retarded the progress of medical science?"

Minute and definite instruction was here given the students as to the method and time of making observations and of preserving facts for future analogy. The magnitude of the field they have to cultivate was briefly referred to, and the usual apology, *want of time*, made by medical men, for neglecting records of important facts and cases, was shown to be incompetent excuse, by the example of Prof. Simpson of Edinburgh, who economized fragments of time in a wonderful degree, and accomplished more professional business than any other man in Europe. Proper methods of investigation and observation with directions for systematic record were introduced and enforced, with the assurance, that if it is ever advantageous to think rightly and observe correctly, it must be so while young, and the mind not preoccupied with theories, but fresh and ductile.

Upon the point of the importance of midwifery and its claims to primary attention, with its rank in the departments of medical science it was freely admitted that chemistry, animal and vegetable; anatomy, special, general and morbid, with physiology, do claim in point of time, the first attention. "These special sciences lay the foundation, the groundwork of practical medicine. They form the basis upon which the fabric is to be created; indeed they are the material of which the superstructure is composed; yet it is apprehended that those only who form their opinions without reflection will deem obstetric science easily acquired or of slight moment. Midwifery has become a science, as well as an art, and it is a laborious task, to thoroughly and satisfactorily learn this branch of medical science."

"During the last century Smellie, the most celebrated London Obstetrician of his day, kept suspended from his office a sign with these words: 'Midwifery taught here for five shillings.' From this fact, some idea may be formed of the amount of learning which he considered requisite for the practice of this, the most important of all the departments of medicine. How changed at the present day. It now comprises within its limits a vast amount of anatomical and physiological acquisition, and requires familiarity with an immense range of therapeutical applications; embraces much of what might be called pure surgery, of a most delicate and difficult character, and is a superstructure on the general base of the other medical sciences. It demands all that the general practitioner ever attains to, and beyond that, much which is peculiar to its own sphere. The important researches made by physiologists and microscopists within the last few years, have completely revolutionized this department of medicine, and lead to the detection of the nature and causes of those affections and diseases which it is necessary for us to comprehend, that we may the better exercise upon them the high ministry of our art."

"Again, there is another branch of this department which has been greatly enriched within the last few years, and which demands your studious consideration. The diseases incident to female organization are becoming more and more numerous; and more and more complicated in their pathology and diagnosis, as well as difficult in treatment, with the advancement of civilization and the extension of luxurious habits, and I hesitate not to assert the belief, that more progress has been made in the diagnosis, pathology and treatment of the affections of the organs connected with the generative functions of the female, during the last ten or fifteen years than in any other department of medical practice."

We must not continue longer our abstract or extracts, but must conclude by quoting the closing paragraph of the Lecture.

"Finally, gentlemen, what do we ascertain to be the science, and what the practice of midwifery that it should be the last on the long list of medical and chirurgical attainments? Whom doth it concern? Can it be of little import to one-half the race that their peculiar nature should be investigated and their diseases especially considered? Is it a small matter to mitigate the pains and agonies of the parturient female? Is it a matter of trifling moment to perform the most *delicate* operations, where the lives of *two* human beings are dependent upon the skill with which it is executed? Is it a small matter, even though life be saved, that the surviving female should not be left in so lacerated and mutilated a state through ignorance or unskillfulness that relief by death itself were a boon? And in the language of another I would add, is it a small matter to deal with the most deadly of epidemics—and to deal skillfully and successfully—to baffle the activity of the most subtle poisons, to keep alive the hearth-fire, to uphold the family altar, which but for us is abandoned and broken down, leaving only the monuments of its beauty and holiness in hearts that are broken, and tears that flow unceasingly?

No, gentlemen, the response is ready from every heart; the resolution is already formed; the solemn purpose taken; that your whole duty shall be performed in this important department, both in the preparation whilst within these halls, and in its faithful execution throughout your future career, when the temporal welfare of whole families shall be entrusted to your care and depend upon your skill.

And though I cannot promise you pecuniary riches, or luxurious ease, I have no hesitation in saying that a far richer reward awaits you, in an approving consciousness of duties deliberately assumed, and honestly discharged; in the gratitude of those whose lives you have saved, or whose sufferings you have alleviated; or, who regard you as the happy instrument in the hands of an overruling Providence of preserving the lives of offspring more precious than their own; and, if pursued with right motives, and a religious trust, a fadeless inheritance.

